# Using Workflows to Explore and Optimise Named Entity Recognition for Chemistry

**DOI:** 10.1371/journal.pone.0020181

**Published:** 2011-05-25

**Authors:** BalaKrishna Kolluru, Lezan Hawizy, Peter Murray-Rust, Junichi Tsujii, Sophia Ananiadou

**Affiliations:** 1 National Centre for Text Mining, Manchester Interdisciplinary Biocentre, University of Manchester, Manchester, United Kingdom; 2 Unilever Centre for Molecular Informatics, University of Cambridge, Cambridge, United Kingdom; Wellcome Trust Sanger Institute, United Kingdom

## Abstract

Chemistry text mining tools should be interoperable and adaptable regardless of system-level implementation, installation or even programming issues. We aim to abstract the functionality of these tools from the underlying implementation via reconfigurable workflows for automatically identifying chemical names. To achieve this, we refactored an established named entity recogniser (in the chemistry domain), OSCAR and studied the impact of each component on the net performance. We developed two reconfigurable workflows from OSCAR using an interoperable text mining framework, U-Compare. These workflows can be altered using the *drag-&-drop* mechanism of the graphical user interface of U-Compare. These workflows also provide a platform to study the relationship between text mining components such as tokenisation and named entity recognition (using maximum entropy Markov model (MEMM) and pattern recognition based classifiers). Results indicate that, for chemistry in particular, eliminating noise generated by tokenisation techniques lead to a slightly better performance than others, in terms of named entity recognition (NER) accuracy. Poor tokenisation translates into poorer input to the classifier components which in turn leads to an increase in Type I or Type II errors, thus, lowering the overall performance. On the Sciborg corpus, the workflow based system, which uses a new tokeniser whilst retaining the same MEMM component, increases the F-score from 82.35% to 84.44%. On the PubMed corpus, it recorded an F-score of 84.84% as against 84.23% by OSCAR.

## Introduction

Text mining for the domain of chemistry is a very challenging task because of the several semantic and syntactic styles in which domain texts are usually expressed. Different aspects such as named entity recognition (NER), tokenisation and acronym detection require bespoke approaches because the complex nature of such texts [1]–[5]. Chemical compounds such as:

17-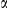-hydroxy-16-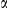-methyl-3,20-dioxopregna-1,4-dien-21-yl acetate

P(Cy)3

1-cyclopropyl-6-fluoro-4-oxo-7-(piperazin-1-yl)-1,

4-dihydroquinoline-3-carboxylic acid hydrochloride

illustrate the complexity of the mining task. Typical word delimiters such as spaces, brackets, hyphens and commas cease to bear the same meaning as in a natural language. As a consequence, the normal text mining approaches such as tokenisers, part-of-speech (POS) taggers and parsers will need to be re-calibrated for this domain as already done for other domains such as biochemistry, biomedicine *etc.*, [6], [7].

In the chemistry domain, researchers have presented a few successful approaches to handle some tasks such as named entity recognition [8]–[13]. However, these approaches usually require reconfiguring and sometimes rewriting everytime a new training corpus or dictionary is released [14], [15]; typically this could be due to different data format or additional information in the new resource. For example, if the new resource is in a different format, the whole system or at least a part of it may need to be rewritten. With the growing number of freely available resources such as Chemspider (http://www.chemspider.com/), Chemlist [14] and [5], [16]–[18] *etc.*, the ability to reconfigure the systems becomes more acute. Such reconfiguring takes time and the subtle changes in the throughputs of these components, which may seem innocuous, could result in the lowering of the net performance of a system; this could be a direct consequence of a suboptimal composition of the workflow. Therefore, it is imperative to configure the optimal set by exploring the various manifestations of the different components [19]. To be able to arrive at an optimum combination of components, one has to substitute one component for another in a workflow and then assess if the performance has indeed improved. This warrants an understanding of inter-component relations working together as a system. It would also be desirable if components using different machine learning techniques could easily be replaced to observe differences in performance. This ability to reconfigure an approach has the advantage of allowing scientists to concentrate more on science rather than format conversion and code refactoring. Usage of workflows for chemistry and its related disciplines has been pursued very actively in the community [20]–[23]. Thus, there is already good familiarity, if not expectation, of this methodology. For the experiments discussed in this paper, we implement reconfigurable workflows that are interchangeable by *drag-&-drop* on the graphical user interface. To do this we employ U-Compare [24]: an open UIMA-based [25] framework (http://incubator.apache.org/uima/ [26]) which allows shareable components, using a common type system, to be used together to form different workflows. In doing so, we also design an interoperable type system for UIMA-compliant systems.

**Figure 1:**
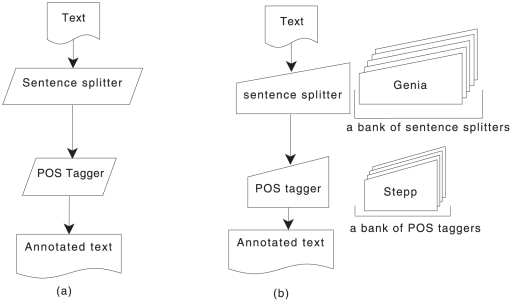
Showing a normal workflow and a reconfigurable workflow as can be built by using U-Compare.

Figure 1 (b) illustrates the composition of a reconfigurable workflow system, wherein one component can be substituted by another component from a repository.

U-Compare framework provides a platform for reconfigurable workflow experiments. Its UIMA-based framework provides the necessary component repository, consisting of several shareable components such as Genia tagger [7], Stepp tagger [27] and OpenNLP [28] sentence splitters, for other UIMA-based components. This extensive repository readily allows for several combinations of components as workflows.

As a consequence, we use a set of individual components to handle the different aspects of text mining, which together form a workflow.

## Related work for workflows

The use of several individual components to construct workflows is quite prevalent amongst the scientific community with interdisciplinary sciences [29]. Bespoke workflows have been employed in several domains such as bio-informatics and earth sciences. Some studies have introduced workflow tools as a Lego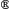-like setup [30], wherein several simple components form a complex workflow which can be easily deployed, modified and tested without the overhead of implementing it into a monolithic application. Taverna [31] is such a workflow management suite for building scientific workflows which offers *loosely-coupled* services. Kepler [32] is another workflow management system for designing, executing, processing and sharing scientific workflows. The workflows in this context are directed graphs where the nodes represent components, the edges represent data paths along which data and results can flow between components.

There are also commercial products which provide environments to create and manage workflows. Pipeline Pilot [33] is an example of a commercial application that combines workflows with data analysis to represent information visually for informatics and scientific business intelligence needs. Pipeline Pilot has been extended to track bibliography in chemistry literature using a web-based graphical user interface [34].

The UIMA platform introduced a new framework for developing shareable components into a repository. Mellebeek *et al.* show the usage of UIMA and text mining applications for curation purposes in the domain of bio-informatics [35]. In doing so, they demonstrate the possible synergy from a combination of diverse expertise in biology, computer science and linguistics. Their application was fundamental to the development of a successful curation tool. The U-Compare [36], based on the UIMA Framework, is an integrated text mining system which provides a graphical user interface for easy *drag-&-drop* workflow creation. It has built-in tools for evaluation and visualisations of components and also has a number of syntactic and semantic tools to generate workflows. Kano *et al.* showed the advantages of using workflows in U-Compare framework by developing a protein-protein interaction extraction system [37].

Our paper presents a similar workflow to [37] but in the domain of chemistry. As a first step, we used Oscar3 [38] to extract chemical named entities from the literature. Subsequently, we segregated Oscar3 into separate components. Townsend *et al.* have developed a methodology and a workflow (CHIC) for the automatic semantic enrichment and structuring of legacy scientific documents by using Oscar3[38]. U-Compare has a plug-in for Taverna [37] which implicitly means the workflows discussed here can be ported to Taverna, which increases the audience and applicability of our workflows.

In the area of chemistry and text mining, Wilbur *et al.* employed two approaches with an aim of separating chemical terms from non-chemical terms [39]:

1. thesaurus-based lexical text analysis using chemical patterns
2. Bayesian classification using n-grams

They found that the Bayesian approach had an overall classification accuracy of 97%, while the thesaurus-based method had an accuracy of 84%. While the work by Wilbur *et al.* operates on individual words (or entities) based on thesaurus-style lists [39], the work described here processes full papers (and abstracts), tokenizes them for analysis and classifies chemical compounds found in the text.

## Materials and Methods

In this paper, our principal task was to elicit chemical compounds from free-flowing text in the chemistry literature. We have used the Sciborg [40] and PubMed [41] corpora for this task.

### Sciborg Corpus

This corpus was compiled as part of the Sciborg project [42]. It consists of 42 articles (full papers) published in the chemical literature which were provided by the Royal Society for Chemistry (RSC). It was curated for linguistic analysis by [41]. This corpus was split randomly into two groups of 14 and 28 papers, such that they form two disjoint testing and training sets respectively. MEMM models (discussed later in paper) were trained on the set of 28 papers having 4102 manually annotated chemical compounds and the 14 papers were used as a test set. The test set was hand-annotated by three chemistry experts and an inter-annotator agreement (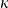) of 0.91 was observed on this set.

### PubMed Corpus

This corpus was compiled for linguistic analysis by Corbett *et al.* [41]. It had 500 abstracts from the PubMed [43] collection. This corpus was randomly split into 400 and 100 abstracts for training and test sets respectively. MEMM models were trained on the 400 abstracts consisting of 4048 annotations of chemical compounds. The test set was hand-annotated by one expert.

### Models

For the MEMM-based component of our approach we experimented with two models,

- *chempaper-M*: trained on Sciborg training data (28 papers)
- *pubmed-M*: trained on 400 PubMed abstracts

### Overview of Oscar3

**Figure 2:**
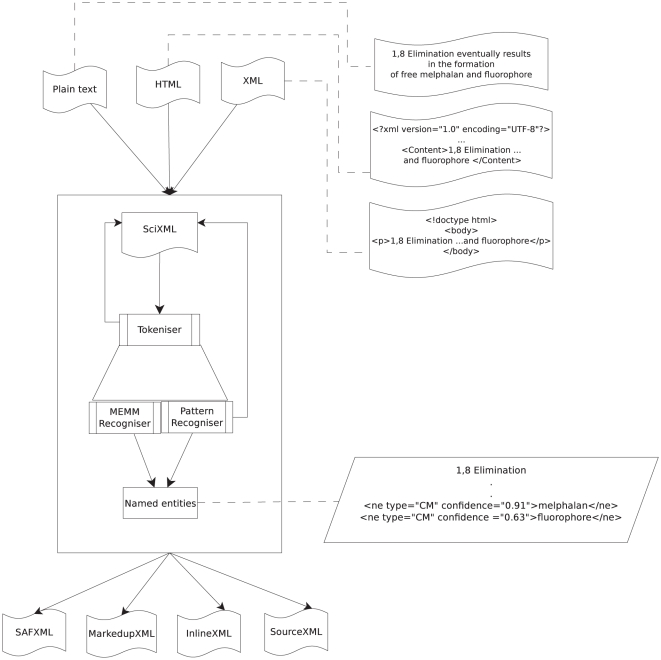
The original architecture of Oscar3.

Oscar3 is an open extensible system for the automated annotation of chemical entities in scientific articles [9]; it was created as part of the Sciborg project [40]. The overall architecture of Oscar3 is shown in Figure 2 and the individual components are discussed below:

#### SciXML

SciXML is the interface used when working with Oscar3, all forms of input (such as XML, HTML and plain text) are converted into this format before any processing is done. It is a form of XML markup used for providing logical structure to scientific papers. Further information about SciXML and its schema can be found in [40].

#### Tokeniser

The tokenisation with Oscar3 is chemistry specific; chemical names are fragile to common methods of tokenisation as they contain potential inter token and intra token characters such as space, hyphens, brackets and comma. The tokeniser here also refers back to the SciXML document to store information about the start and end points of a token as well as its content. For example, some of the tokens in data are:

aztreonam
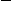
Metallo-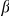-lactamase

Cu2
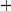


C2(MONO)

C
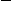
O/C
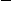
N

Zn
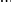
O3S(monobactam)

#### Chemical Entity Recognisers

Oscar3 contains two types of chemical entity recognisers, each producing a list of named entities as an output containing chemical annotations, such as token, types and likelihood scores (where applicable).

#### Pattern Recogniser

This recogniser was initially used before the machine learning component was introduced. It uses deterministic finite state automata alongside ontologies (such as CHEBI [44]), dictionaries and n-gram models to recognise the named entities. As it relies on the regular expression based rules, it does not use any mathematical models for classification.

#### MEMM Recogniser

This recogniser uses MEMM and character level n-grams to recognise chemical entities based on their likelihoods. The MEMM was trained using the annotated corpora discussed earlier. Corbett *et al.* reported an F-score of 80.7% for a model trained on Sciborg and PubMed training sets at a confidence threshold of 0.3 [41].

#### Why the new Oscar?

Oscar3 is an efficient annotation tool and is widely used within the chemistry domain. However, the architecture is rigid and, due to its dependency on the SciXML format and the interdependency within the different components, it is difficult to modularise and it does not readily adapt to new and emerging trends in annotation and corpora. This puts a limitation on enabling and refactoring reusable components.

### Oscar3 as a Workflow of Reconfigurable Components

**Figure 3:**
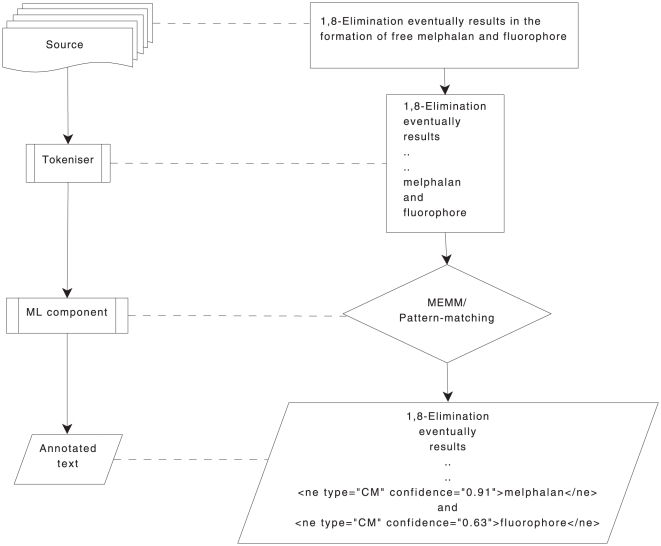
Oscar3 refactored as a workflow of different components.

Conceptually, Oscar3 [9] is a named entity recogniser which classifies tokens into chemical entities based on either likelihoods or a pattern match. Therefore, Oscar3 was divided into the following components as shown in Figure 3. This is just one of the many possible manifestations of the workflows; other configurations such as different tokenisers and components implementing machine learning techniques can be easily accommodated to make a new workflow.

- A tokeniser: This tokeniser is a white-space delimited, word eliciting component which reads content from files in text, XML or HTML and yields tokens similar to the syntactic token of the U-Compare type system [45]. It must be noted here that any tokeniser that yields the syntactic tokens for a given source file can be used as the first stage of the Oscar workflow.
- A MEMM Component: trained on two chemistry-specific corpora (as mentioned in the data section).
- A Pattern matching Component: based on a finite state automaton driven regular expression matcher; the rules of which were designed after several observations of the training data. As a consequence, this component does not use any statistical models for classification purposes.

**Figure 4:**
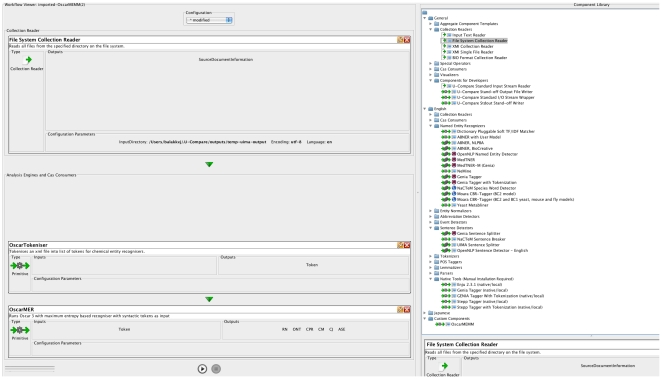
U-Compare view of Oscar workflow. Right side of the figure shows a workflow made from the Oscar components shown on the left.

Shown in Figure 4 is one of the workflows (left in the figure) using three components (right in the figure): a file system component to read the files which are then split into individual tokens by the OscarTokeniser component which subsequently feeds into the OscarMER component to classify the tokens into chemical names.

## Results

The experiments described earlier with Oscar3 and its refactored version were designed to study the effect of tokenisation on chemical element identification. We present the results of modularising Oscar3 and compare it with the existing version. Also, to present the robustness of the workflow, we compare the performance of Oscar3 on the corpora described in the data section.

### Reconfiguring Oscar3: a confidence-driven approach

**Figure 5:**
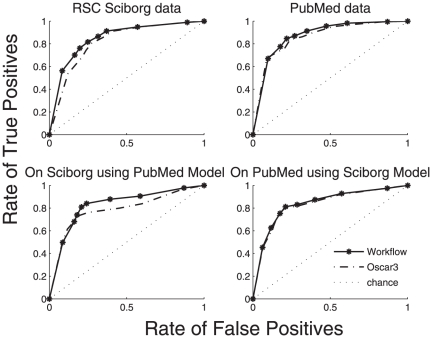
ROC curves comparing the performance of various Oscar variants. In all the four different experiments, Oscar workflow has a slight edge over the Oscar 3 variant.

The machine learning components used by the two variations of Oscar3 (Oscar3 stand-alone version and Oscar workflow) yield a confidence score, which is a likelihood estimate (see [41] for more details), to show the confidence in that annotation. In order to arrive at an optimum threshold for each of the corpora, we have plotted the ROC (A ROC curve is a receiver operating characteristic which plots the rate of true positives against the rate of false positives.) curves for each of the data sets. Shown in Figure 5 are the ROC curves for different combinations of data sets and Oscar3 variants.

### Oscar vs. Oscar: One Variant against Another

As described earlier, currently there are two types of named entity recognisers, a MEMM-based and a pattern matching one. The MEMM-based versions were tested with two models; *chempaper-M* and *pubmed-M*.

The results are presented in terms of percentages of precision (P), recall (R) and F score (F). Table 1. shows the overall performance of both the variants of Oscar3 on Sciborg test data. The MEMM-driven systems were tested using both models (*chempaper-M* and *pubmed-M*) which are trained on Sciborg and PubMed training data respectively.

**Table 1:** Performance (%) of different variants of Oscar on Sciborg test data using the models trained on Sciborg data and PubMed data.

Variants on Sciborg	Model used	
	*chempaper-M*	*pubmed-M*
Oscar3 (MEMM)	P 88.24	74.76
	R 77.19	65.18
	F 82.35	69.64
Oscar workflow (MEMM)	P 90.31	80.19
	R 79.29	71.22
	F 84.44	75.44

Table 2. shows the performances of Oscar3 with pattern recogniser (Oscar3 (PAT)) and as a workflow with pattern recogniser (Oscar workflow (PAT)) on the Sciborg test data.

**Table 2:** Performance of different Oscar pattern recogniser versions on Sciborg.

Variants on Sciborg	Scores (%)
Oscar3 (PAT)	P 70.43
	R 67.42
	F 68.89
Oscar workflow (PAT)	P 74.11
	R 73.68
	F 73.90

As described in Corbett *et al.* [41], Oscar3 can be tuned to filter out some false positives (Type I) errors based on a confidence score derived from the logit scores (see [41] for more details). At a confidence score of 0.42, Oscar3 as a workflow with MEMM recorded an F-score of 84.84% while Oscar3 with MEMM recorded 82.35% on the Sciborg data. It is also noteworthy that although the pattern recognition variants were less accurate than their MEMM counterparts, the workflow variant still outperforms its monolithic parent.

Table 3 shows the performance of the Oscar3 variants when used on the PubMed test set against models trained on Sciborg and PubMed training data sets. It can be observed that a different tokeniser gives an extra boost of 0.61% (84.84 by the workflow MEMM variant as against 84.23 by the Oscar3 variant) whilst retaining the same machine learning component and the model.

**Table 3:** Performance of different variants of Oscar on PubMed test data using the models trained on Sciborg data and PubMed data.

Variants on PubMed	Model used	
	*chempaper-M*	*pubmed-M*
Oscar3 (MEMM)	P 75.28	89.04
	R 63.42	79.91
	F 68.84	84.23
Oscar workflow (MEMM)	P 75.06	85.66
	R 64.58	84.03
	F 69.43	84.84

Table 4 shows the performance of pattern-recognition based variants of Oscar3 and Oscar3 workflow on the Pubmed data. Although having lower scores than the MEMM variants, Oscar workflow (PAT) outperforms the Oscar3 (PAT) by 1.7%.

**Table 4:** Performance of different Oscar pattern recogniser versions on Pubmed.

Variants on Pubmed	Scores (%)
Oscar3 (PAT)	P 44.22
	R 58.24
	F 50.27
Oscar workflow (PAT)	P 45.64
	R 60.35
	F 51.97

Wren used the single-order Markov models to distinguish between chemical and non-chemical terms on Medline [46] corpus with an average precision of about 82.7% [10]. The work described here uses maximum entropy Markov models on 2 different corpora: Sciborg and PubMed. For this corpus, our approach recorded a precision of 90.31% and a recall of 85.66%. As the work by Wren ([10] ) and our approach vary on the corpora and methodology, we do not see it fair to compare head-to-head; however, our system does perform as well, if not better.

## Discussion

To observe the efficacy of workflows, we have used two sets of workflows, one each in pattern recognition based variant and the MEMM variant. As shown in Tables 1 and 2, the workflow variants of Oscar3 achieve better performance the two variants.

On the Sciborg, the workflow-based MEMM model achieved an F-score of 84.44% as opposed to 82.35% for Oscar3. We observed that this increase was due to removal of the dependency on SciXML conversions within the workflow.

A reconfigurable approach enabled us to identify the erroneous (or underperforming) component and relate some of the errors to severe dependency on SciXML conversions, when using *chempaper-M*. We infer that there was a net increase in false positives due to the noise in several inter-conversions of formats in SciXML. It should be noted here that the dependence of Oscar3 on SciXML was due to them both being part of the Sciborg project. This dependency could make it difficult to adapt to newer corpora. It was observed that the new tokenisation identified more chemical words such as


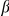
-lactam
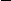
zn2+

bis-monodentate

gold
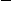
sulfur

which automatically led to a decrease in false positives (Type I errors). It also avoided wrongly tokenizing a few words such as,

diimine

mono-bidentate

which were subsequently omitted as non-chemistry words by the Oscar3 but accurately identified by the workflow variant. In this example, the complete chemical was ruthenium (ii) diimine, but Oscar3 returned only ruthenium(ii) as CM, whilst the workflow version got the complete entity. by the Oscar3 but accurately identified by the workflow variant. This led to fewer false negatives (Type II errors) and hence a better recall.

As the machine learning classifiers and the models they used were exactly the same for all experiments, we infer that tokenisation on the Sciborg test avoided partial entities for recognition and this helped reduce both Type I and Type II errors.

**Figure 6:**
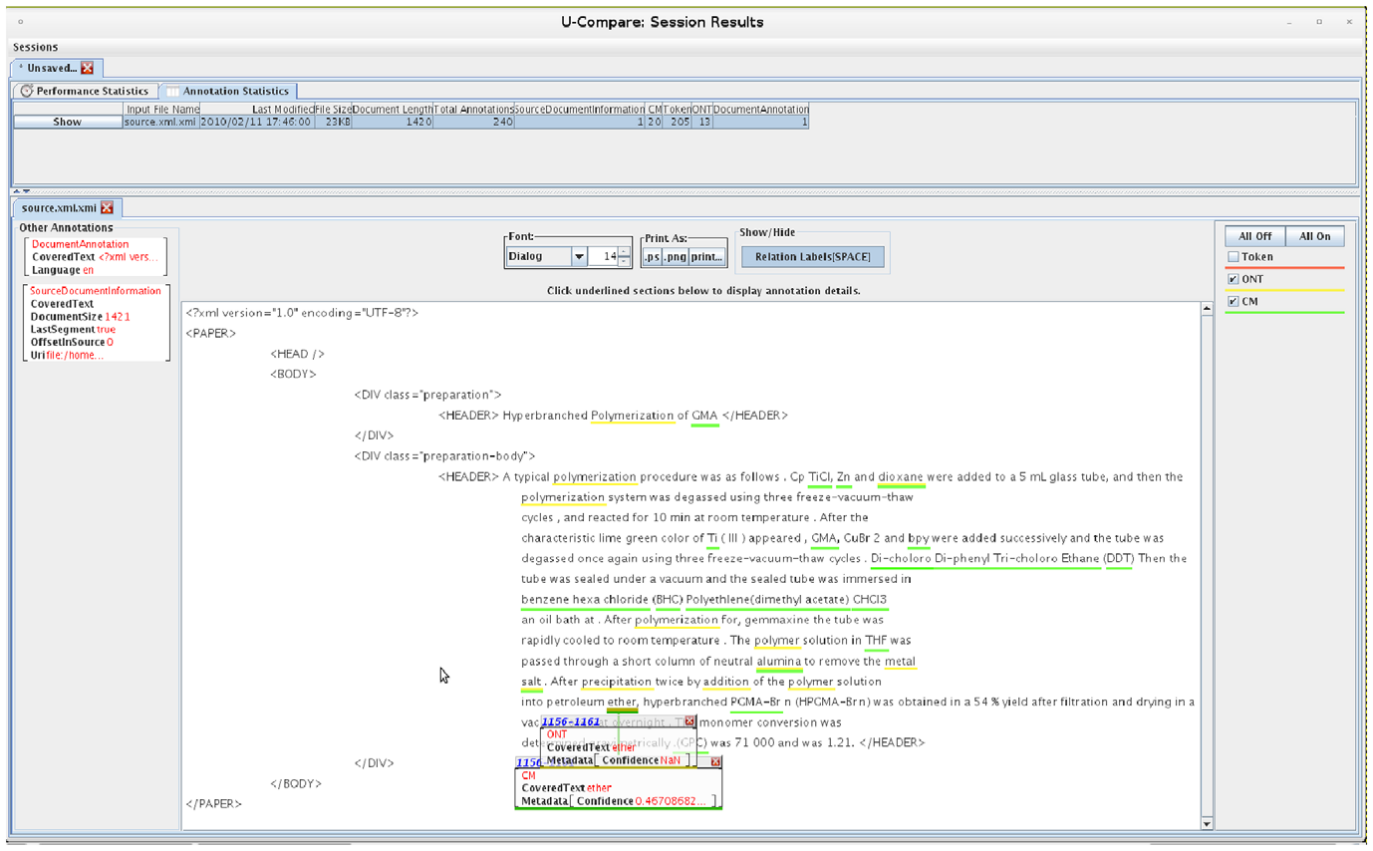
U-Compare output for a test document. Chemical names (underlined) as identified by the MEMM-based workflow.

Figure 6 shows the chemical names as annotated by the Oscar workflow in the U-Compare framework. When these entities (which are underlined) are clicked, more information about the entity such as confidence scores, metadata *etc.* is available to the user.

Table 3 shows a decrease in precision of 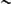
3% with an increase in recall of 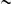
4% for the PubMed test data. Perhaps, this could be attributed to the possible shortcomings in the ability of the new tokeniser to adapt to the biochemical entities; we are working on enhancing the tokeniser to suit multiple domains where chemistry plays an important role.

The current version of Oscar3, which can be downloaded from http://sourceforge.net/projects/oscar3-chem/, had an F-score of 82.35% on Sciborg as against 80.7% achieved by [41]. This could be due to the fact that [41] used a 3-fold cross validation, whilst we used only 1 combination. The usage of one training set and one test set, instead of multi-fold cross validation, was guided by the focus of our paper, namely: the advantages of workflows for text mining in chemistry. Also, as described in the Data section, the test set comprised of 14 full papers, manually annotated by three experts, whilst, the training set was annotated by a single expert. We conjecture that this test set had enough data points to support our inferences.

On the Sciborg (Table 1), the pattern-recognition based workflow achieved a precision of 66.32% while the Oscar3 using the pattern recognition module achieved a precision of 44.65%. Again, it seems the only difference between the two variants was the tokenisation which stems from issues relating to SciXML conversions. This could be perceived as an example of having an optimal combination in a workflow to derive a better performance.

The results indicate the success of workflows described in our experiments discussed earlier. Currently, we are in the process of converting implementations of other machine learning algorithms such as Conditional Random Fields (CRF) into the U-Compare framework. This will enable us to compare the performance of different algorithms on data sets. Every time a new annotation scheme is announced, it obliges the existing applications to adapt, sometimes subtly and at times extensively. We have shown that a reconfigurable system (or application) is better for such adaptation.

### Conclusions

We have shown that, using a reconfigurable workflow, it is possible to assess different components in a system to elicit the best combination. As a consequence, it helps users to focus less on system implementation issues. Using these workflows, we studied the impact of using different tokenisation techniques on the task of named entity recognition in chemistry. The potential for expanding the scope of inter-component analysis is immense and more so, with complex systems involving several components. We have demonstrated the impact of tokenisation in recognising complex named entities in chemistry, wherein a named entity may contain two, three or even four words with numerals, Greek letters, punctuation marks, *etc.* Work is currently underway to make a CRF component so that one can freely replace MEMM models with a CRF model and thus benefit from a pool of machine learning algorithms for various tasks, named entity recognition being one of them. We are also working on workflows to combine a set of taggers and named entity recognisers for application in the domain of chemistry, biochemistry and biological sciences [47].
